# Immediate and Short-Term Effects of Nature-Based Immersive Virtual Reality During Dry Needling: A Single-Blinded Randomized Controlled Trial

**DOI:** 10.3390/jcm15145683

**Published:** 2026-07-20

**Authors:** Rodrigo Martín-San Agustín, Alberto Gadea-Blázquez, Elena Millán-Magariños, Adrian Escriche-Escuder, Borja Tronchoni-Crespo, Javier Guerra-Armas

**Affiliations:** 1 Clinimetry and Technological Development in Therapeutic Exercise Research Group (CLIDET), Department of Physiotherapy, University of Valencia, 46010 Valencia, Spain; rodrigo.martin@uv.es (R.M.-S.A.); e.millanmagarinos@edu.gva.es (E.M.-M.); borja.tronchoni@uv.es (B.T.-C.); jguerra@ufpcanarias.es (J.G.-A.); 2 Department of Physiotherapy, University of Fernando Pessoa Canarias, 35450 Las Palmas, Spain

**Keywords:** musculoskeletal pain, virtual reality, dry needling, pain management, rehabilitation

## Abstract

**Objectives:** This study aimed to investigate the effects of nature-based immersive virtual reality (IVR) applied during dry needling (DN) on pain experienced during the procedure and on immediate and short-term post-intervention outcomes, compared with traditional DN, with a primary focus on immediate post-intervention pain intensity. **Methods:** In this single-blinded randomized controlled trial, participants were randomly assigned to the control (DN) or experimental (DN + IVR) group. Eligible participants were healthy individuals with identifiable latent myofascial trigger points (MTPs) in the quadriceps muscles. The primary outcome was pain intensity, measured using a numerical rating scale (NRS), immediately after the intervention. Secondary outcomes included pressure pain threshold (PPT) and pain intensity at follow-up time points (1, 6, and 24 h). Linear mixed-effects models were used to analyze the effects of group, time, and their interactions. **Results:** A total of 40 participants (control group, n = 21, 21.7 ± 3.1 years, 15 males; experimental group, n = 19, 22.4 ± 3.7 years, 11 males) were included. The DN + IVR group showed significantly lower pain intensity immediately after the intervention than the DN group (*p* = 0.032). Pain intensity decreased over time in both groups (*p* < 0.001), with no significant intergroup differences at later time points (all *p* > 0.05). For PPT, a significant effect of time was observed (*p* < 0.001), but no significant group × time interaction was observed. **Conclusions:** Nature-based IVR may reduce immediate procedural pain associated with dry needling, although these effects are not sustained over time. IVR may be a useful non-pharmacological strategy for pain relief during invasive physiotherapy procedures.

## 1. Introduction

Myofascial pain syndrome (MPS) is a non-inflammatory disorder of musculoskeletal origin associated with muscle pain and stiffness and has a lifetime prevalence of approximately 85% in the general population [1]. MPS involves palpable hyperirritable nodules in skeletal muscle fibers (myofascial trigger points, MTPs), although its exact pathophysiology remains unknown [2]. Although approaches for the management of musculoskeletal pain associated with MPS vary, dry needling (DN) has been increasingly used by physiotherapists to treat musculoskeletal pain in recent years [3]. Evidence shows that dry needling (DN) effectively reduces pain in musculoskeletal conditions [4,5].

DN is a minimally invasive therapeutic procedure that involves the insertion, through the skin, of a thin, threadlike needle without an orifice into an area of muscle previously described as an MTP without the addition of local pharmacological solutions or agents [4]. The needle is inserted until it causes a local twitch response and is then withdrawn from the muscles. DN, like other invasive physical therapy techniques, is safe as long as it is performed correctly; however, like any physical therapy technique, it can have adverse effects. Indeed, a US survey of physiotherapists (n = 865) reported that minor adverse events were common (39.6% of treatments) during DN [3]. These effects are usually mild and temporary, and serious complications are rare. However, previous studies have reported that the most frequent adverse effects of invasive techniques were pain during the intervention (96.1%) and in the following days (71.1%), as well as mild post-intervention vasovagal responses (80.1%) being inherent to the stimulus generated by the needle [6].

Among the few absolute contraindications to invasive physiotherapy are those who suffer from belonephobia or extreme and uncontrollable fear of sharp objects, such as needles, which can lead to syncope, fainting, or phobic attacks [7]. This contraindication poses a challenge for professionals who use invasive techniques because it renders the procedure impossible. Frequent stress and anxiety associated with needle procedures can cause fear and nocebo effects [8]. To overcome these adverse effects during DN interventions, distraction strategies have been proposed to reduce pain, anxiety, and/or fear in clinical scenarios [9]. Distraction has been extensively studied in both experimentally induced pain and acute and chronic clinical pain, revealing a complex web of cognitive and motivational factors that influence its effectiveness in pain relief. Distraction-based strategies direct attention away from pain using competing demands and involve a process by which the patient’s senses are diverted from the nociceptive stimulus [10,11].

In recent years, research has focused on developing effective distraction methods for needle-related procedures in clinical practice. Among these strategies, immersive virtual reality (IVR) has been shown to be effective in reducing pain intensity, fear, and anxiety during interventions in different clinical settings in both adult and pediatric populations [12,13,14]. IVR has been proposed as a promising multisensory distraction modality, and a previous meta-analysis has confirmed the hypoalgesic effects of IVR on acute and short-term pain in adult and pediatric populations [15]. IVR allows healthcare professionals to effectively influence the pain experience of patients undergoing painful procedures by completely distracting them from nociceptive stimuli. IVR has shown benefits in other painful procedures, such as blood draws, injections, dental procedures, and wound healing [13]. While a significant reduction in reported pain during painful medical procedures has been observed when an IVR environment distracts patients [16], IVR-based distraction strategies for DN interventions have not been previously studied. Therefore, this study aimed to examine the immediate and short-term hypoalgesic effects of nature-based IVR following the DN procedure. Specifically, the primary objective was to investigate the effects of nature-based immersive virtual reality (IVR) applied during dry needling (DN) on pain experienced during the procedure. The secondary objectives were to assess the effects on pressure pain threshold (PPT) and pain intensity at short-term follow-up time points.

## 2. Materials and Methods

### 2.1. Study Design

A randomized controlled trial was conducted at the University of Valencia between September 2024 and February 2025. A two-group repeated-measures design compared immediate and short-term hypoalgesic effects following DN of nature-based IVR, using healthy participants randomly distributed into experimental (DN + IVR) or control (DN without distractors) groups. Participants were randomized by an independent researcher using an online tool (https://ctrandomization.cancer.gov/). Opaque sealed envelopes were used during the assignment process to ensure allocation concealment of the treatment. The study was approved by the Ethics Committee of the University of Valencia (Spain) (3250436) and prospectively registered at clinicaltrials.gov (NCT06448104). This manuscript was reported following the Consolidated Standards of Reporting Trials (CONSORT) checklist [17] (Supplementary Materials).

### 2.2. Population and Eligibility Criteria

Forty healthy participants were recruited via email through the University of Valencia Intranet by a professor from the same university. For inclusion, participants had to meet the following criteria: (I) age between 18 and 45 years, (II) at least one identifiable latent myofascial trigger point in the muscles studied, (III) no musculoskeletal or neural disorder that could alter the results obtained, and (IV) no belenophobia or extreme and uncontrollable fear of sharp objects. Prior to data collection, participants completed an information sheet that included demographic (age and sex) and anthropometric (height and weight) measurements. Participants provided written informed consent prior to their inclusion in the study.

### 2.3. Outcome Measures

The primary outcome of this study was pain intensity during DN, assessed using a 0–10 numerical rating scale (NRS), where 0 represents “no pain” and 10 represents “worst imaginable pain.” Pain intensity was assessed immediately after the intervention, with participants instructed to retrospectively rate the pain experienced during the DN procedure. This reflects the main objective of the study, namely, the evaluation of the immediate hypoalgesic effect of IVR during the procedure.

**Figure 1:**
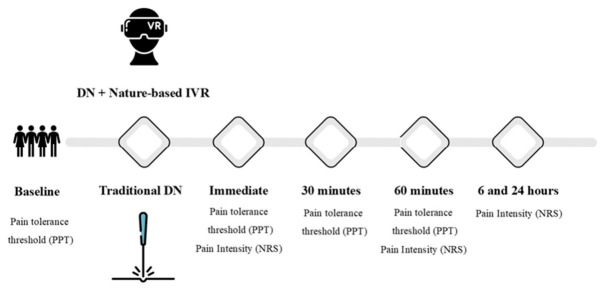
Illustration of the experimental setup and procedures.

The secondary outcomes included PPT and pain intensity at follow-up. PPT was assessed using a digital pressure algometer (Wagner Instruments FDK 20; Greenwich, CT, USA) at the site of the latent myofascial trigger point. A gradual perpendicular pressure of approximately 100 kPa/s was applied until the participant reported the first sensation of pain, and the value was recorded. The mean of three consecutive measurements was used for the analyses. PPT was evaluated at baseline (pre-intervention), immediately post-intervention, 30 min, and 1 h after the intervention. Pain intensity at follow-up was recorded using the same NRS at 1, 6, and 24 h after the intervention to assess short-term effects (Figure 1).

All outcome measures were evaluated by an examiner who was blinded to the participant group. Thus, the examiner responsible for measuring these variables left the room after the pre-intervention measurements. Once the other examiner applied DN with or without IVR, the first examiner entered the room to perform the post-intervention measurement and was responsible for asking about pain via telephone at 1 h, 6 h, and 24 h later.

### 2.4. Procedures

**Figure 2:**
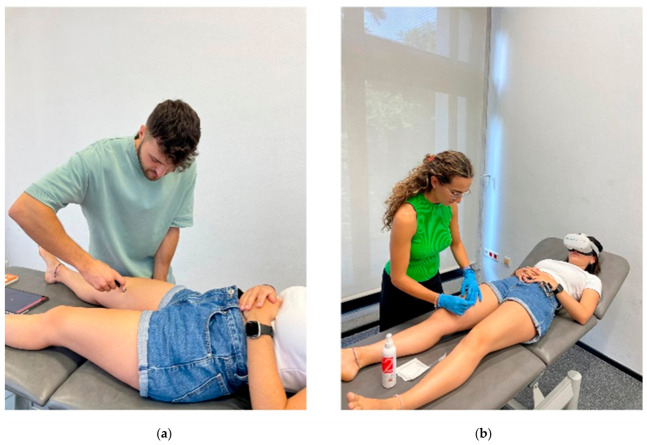
(**a**) Measurement position for pain threshold to pressure; (**b**) dry needling at the previously marked point of the vastus medialis quadriceps.

Both groups underwent a DN procedure based on the Hong technique on the main latent MTP of the vastus medialis quadriceps muscle [18]. This technique consists of repeated needle insertion (rapid entries and exits) into the MTP to obtain as many local spasm responses as possible within the patient’s tolerance [19]. As the participants were healthy subjects, it was applied only to the main latent MTP identified in the dominant lower limb (right side in all participants), based on pain elicited upon palpation, with the participants in the supine decubitus position. Latent MTPs were identified based on the presence of a palpable taut band and a hypersensitive spot within the muscle, along with the elicitation of referred pain upon palpation, in accordance with established consensus-based criteria [18]. Given that the participants were asymptomatic, the absence of spontaneous pain was ensured, and recognition of evoked pain as familiar pain was not required. The depth of penetration varied to reach the MTP of each subject. Needling continued until local spasm responses ceased, with limits set by patient tolerance, a maximum of 20 needle insertions, and one minute of application, following a previous protocol [19]. A 0.30 × 40 mm needle (Acupunt, Barcelona, Spain) was used. Post-puncture manual hemostasis was not performed because this procedure is associated with a reduction in post-puncture discomfort, one of the aspects analyzed in this study. Prior to needling, the skin surface was disinfected using a 2% alcoholic chlorhexidine spray. The researcher who applied the technique wore gloves during the procedure. The participants in both groups underwent the procedure in the same position (Figure 2).

**Figure 3:**
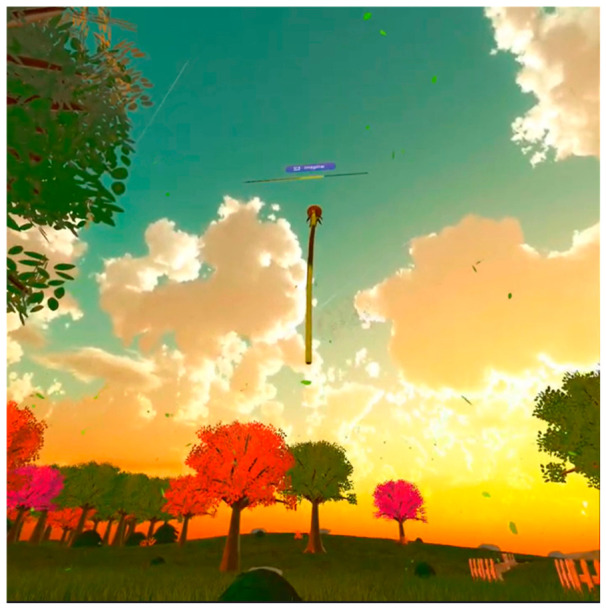
Nature-based immersive VR multisensory environment (Dynamics VR Rehab, Seville, Spain). All images have been reproduced with the permission of the respective company, whereby all rights to these images are owned by the respective rights holders.

In the experimental group, the DN procedure was performed simultaneously with the nature-based IVR distraction software (“Dynamics VR Active” 1.2 (Dynamics VR Rehab^®^, Seville, Spain)) applied through a Meta Quest II VR head-mounted display (HMD) (Meta Platforms, Inc., Menlo Park, CA, USA). The Meta Quest II HMD was used for its accessibility, ease of use, and minimal visual latency. For the IVR condition, commercially available software (Dynamics VR Rehab, Seville, Spain) was used, in which participants were immersed in a nature-based landscape in a meadow with trees. In addition, a guided breathing exercise with visual feedback (such as a dandelion releasing its petals) was included with the instructions “inhale” and “exhale” following a square breathing pattern [20]. The scientific literature indicates that exposure to nature-based VR may have significant effects on anxiety and pain relief, thus promoting a state of relaxation [21]. IVR distraction software has multisensory inputs (vision and sound), high-quality graphics, soft music, and head and controller tracking, allowing for a highly immersive experience for patients. Likewise, user experience factors that have been associated with greater hypoalgesia, such as presence and interactivity, were included (Figure 3). These visualizations began prior to the needling and ended immediately after the procedure was completed. The entire intervention was performed by the same physiotherapist experienced in DN, myofascial pain examination, and IVR throughout the study.

### 2.5. Statistical Analysis

All statistical analyses were performed using the IBM SPSS Statistics software (version 29.0, IBM Corp., Armonk, NY, USA). Participants’ characteristics are summarized as means (standard deviation (SD)), and PPT and pain intensity measurements are summarized as means (SD) and 95% confidence intervals (CIs). The normality of the distribution of PPT and pain intensity was evaluated using the Shapiro–Wilk test.

To account for the repeated-measures design and inter-individual variability, linear mixed-effects models (LMMs) were fitted separately for pain intensity and PPT. Group (DN vs. DN + IVR), time, and their interaction (group × time) were included as fixed effects, whereas a random intercept for each participant was included to account for between-subject variability. Time was treated as a categorical variable in this study.

An unstructured residual covariance matrix was specified to model the covariance among repeated observations within the participants. The models were estimated using the restricted maximum likelihood (REML). Parameter estimates (β) with corresponding 95% confidence intervals (CIs) are reported for all the fixed effects. When significant main or interaction effects were identified, post hoc pairwise comparisons of estimated marginal means were performed using Bonferroni adjustment. Statistical significance was set at *p* < 0.05. An a priori sample size estimation was performed based on feasibility considerations and previous studies.

Given that this was the first study to evaluate pain intensity during and following DN using IVR as a distraction intervention, a formal effect size specific to this scenario was not available. Therefore, the initial sample size was based on previous experimental studies involving dry needling in similar muscle groups, which typically included approximately 18 participants per group [22]. Assuming a potential dropout rate of 10%, a target sample size of 20 participants per group was determined. In addition, to further support this estimation, evidence from VR hypoalgesia studies suggests large effects on pain reduction. A meta-analysis reported a mean effect size of Cohen’s d = 0.90 for VR interventions on pain outcomes [23]. Under these assumptions (α = 0.05, power = 80%), the estimated required sample size would be approximately 36 participants, which is consistent with the sample size included in the present study (n = 40).

## 3. Results

**Figure 4:**
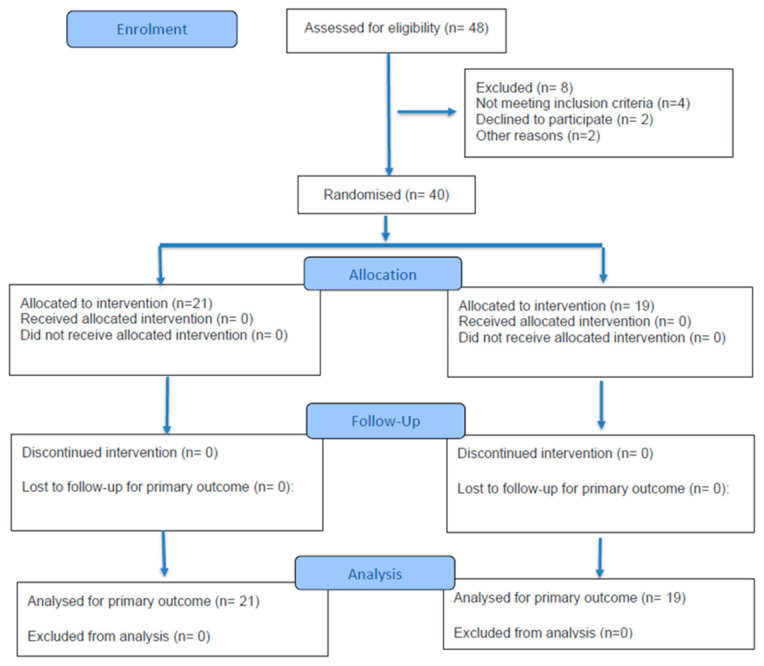
CONSORT 2025 flow diagram.

A total of 40 pain-free individuals (DN group, n = 21, 21.7 ± 3.1 years, 15 male participants; DN + IVR group, n = 19, 22.4 ± 3.7 years, 11 male participants) were included (Figure 4). The groups did not differ in sex, age, body mass, stature, or PPT at baseline.

### 3.1. Procedures

In all participants, a maximum of 20 needle insertions was completed in less than one minute, which determined the endpoint of the intervention in all cases. The number of local twitch responses and the exact duration of the procedure were not recorded; however, the presence of at least one local twitch response was confirmed in all the participants.

### 3.2. Pain Intensity

Table 1 presents the pain intensity values by time point and group. A linear mixed-effects model was used to evaluate the effects of group, time, and their interaction on pain intensity. We observed a significant main effect of time (*p* < 0.001), indicating a reduction in pain intensity over time in both groups. Compared with the 1 h time point (reference), pain intensity was significantly lower at 6 h (β = −1.19, 95% CI [−2.04, −0.35], *p* = 0.006) and 24 h (β = −1.86, 95% CI [−2.70, −1.01], *p* < 0.001) and significantly higher immediately after the intervention (β = 3.55, 95% CI [2.70, 4.39], *p* < 0.001). No significant main effect of group was found (β = −0.01, 95% CI [−0.93, 0.92], *p* = 0.991), indicating no overall difference in pain intensity between the groups across time. A significant group × time interaction was observed at the immediate post-intervention time point (β = −1.34, 95% CI [−2.56, −0.11], *p* = 0.032).

Consistent with this result, the between-group mean difference in pain intensity immediately after the intervention was −1.34 points (95% CI [−2.56, −0.11]), indicating lower pain in the DN + IVR group than in the DN group. At 1, 6, and 24 h, the between-group mean differences were small and not statistically significant. Specifically, we found the estimated differences close to zero, and their corresponding confidence intervals included zero, indicating no meaningful differences between the groups at these time points.

**Figure 5:**
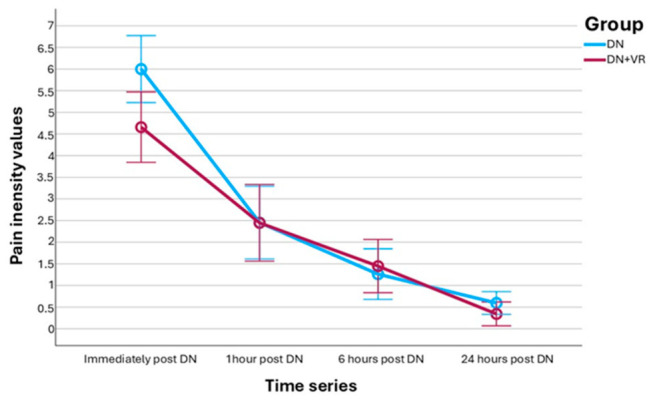
Pain intensity values by moment of measurement and group.

No significant between-group differences were found at 1, 6, or 24 h (all *p* > 0.05), and the corresponding mean differences were small and not clinically meaningful, indicating similar pain reduction trajectories between the groups during follow-up (Figure 5).

### 3.3. Pressure Pain Threshold (PPT)

Table 2 shows the pressure pain threshold values by time point and group. A linear mixed-effects model was used to examine the effects of group, time, and their interactions on PPT. A significant main effect of time was observed (*p* < 0.001), indicating changes in PPT following the DN intervention in both groups. No significant main effect of group was found (all *p* > 0.05), suggesting no overall differences in PPT between the groups across time. The group × time interaction was not statistically significant (all *p* > 0.05), indicating that the changes in PPT over time did not differ significantly between the groups.

**Figure 6:**
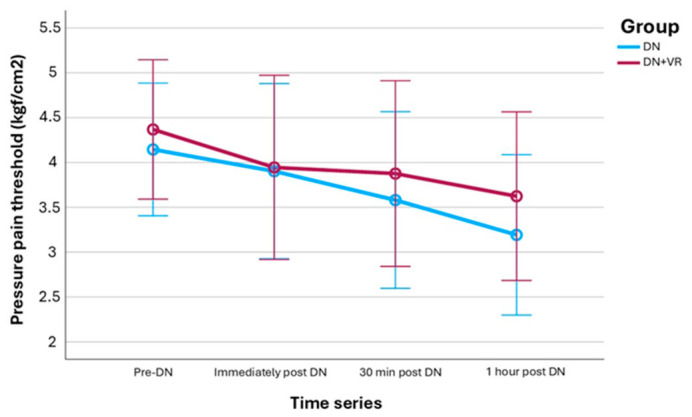
Pressure pain threshold values by moment of measurement and group.

The differences between groups were small at all time points. Immediately after the intervention, the mean difference was 0.04 (95% CI [−1.38, 1.46]), and at 30 min, it was 0.30 (95% CI [−1.15, 1.75]). At 1 h, the mean difference was 0.43 (95% CI [−0.90, 1.76]). All confidence intervals included zero, indicating no statistically significant between-group differences in PPT at any individual time point. Taken together, these findings are consistent with the absence of a significant group × time interaction and suggest that the temporal evolution of PPT was similar in both groups. Table 2 and Figure 6 show the PPT values over time by group.

### 3.4. Adverse Events

No adverse effects were reported beyond the expected discomfort or mild pain during or after the dry needling procedure.

## 4. Discussion

This study aimed to investigate the immediate and short-term hypoalgesic effects of nature-based IVR on different measures of pain modulation in acute procedural pain related to DN, with a primary focus on pain intensity during DN. The main finding was that participants in the DN + IVR group reported significantly lower pain intensity immediately after the intervention than those in the DN group. However, this effect was not maintained over time, as no significant between-group differences were observed at the subsequent follow-up time points. Additionally, no consistent between-group differences were observed for PPT. These findings suggest that nature-based IVR reduces pain intensity during DN, offering a promising approach to reducing acute procedural pain associated with needle-related procedures.

Our results are consistent with those of previous studies reporting significant reductions in self-reported pain (−1.74 NRS) during invasive interventions, such as intramuscular injections, compared to control conditions [24]. Similarly, a study examining VR during arteriovenous needle insertion showed a statistically significant reduction in pain (−2.43 NRS) [25]. The magnitude of pain reduction observed in our study and previous research approaches the minimum clinically important difference (MCID) in pain intensity established at 2.0 on the numerical rating scale (NRS) [26], suggesting a potentially meaningful clinical effect. Hence, our findings provide a clinical alternative to minimize procedural pain during DN, with a large effect size observed for pain intensity (d = 1.75). Acute pain during DN may limit its use in many patients [6]. In addition, fear of needles is common in both children and adults and may lead to increased anxiety during procedures or interference with clinicians’ abilities to conduct procedures [7]. IVR is expected to become even more effective and accessible in the near future, and the rapidly evolving technology [27,28] offers enormous clinical potential for minimally invasive physiotherapy interventions such as DN, percutaneous neuromodulation, or percutaneous electrolysis [29].

While chronic pain conditions are often associated with impaired conditioned pain modulation (CPM) [29], healthy individuals tend to exhibit more efficient endogenous pain modulation, which may influence the magnitude of IVR-induced hypoalgesia [30,31]. IVR-based distraction has shown positive short-term effects, particularly in acute or experimental pain [13,15], whereas its effectiveness in chronic pain appears to be more limited [32]. Therefore, given the nature of our sample, the findings should be interpreted cautiously when extrapolating to clinical populations, and further research is needed.

In this study, no significant between-group differences were observed in PPT, despite differences in self-reported pain immediately after the intervention. While IVR-induced hypoalgesia refers to reductions in perceived and/or evoked pain, our findings suggest that different mechanisms may underlie the changes in subjective pain perception and mechanical sensitivity. Although the exact mechanisms of action of DN have not yet been unraveled, a wide range of local, peripheral, and central mechanisms have been proposed [33,34]. Our findings could be explained because the mechanisms of action underlying the immediate effect of IVR may involve a distraction effect [35], but the local hypersensitivity in the DN-treated area is likely mediated by peripheral sensitization phenomena and biochemical changes [33,36]. Given that the distracting effects of IVR may occur without altering DN’s physiological effects, it would explain the trajectories of pain recovery over time in both groups. Conversely, these immediate and temporary effects of DN have also been observed in the significant increase in sympathetic nervous system activity for up to 18 min. Later, 21 min after the intervention, the values of autonomic response activity returned to a non-significant difference compared to the baseline value [37]. This may explain the 1 h PPT increase observed in the IVR group, similar to the study by Colloca et al. [38], where IVR seemed to modulate sympathetic arousal. These findings prompt questions regarding the immediate and short-term mechanisms of DN and IVR-induced hypoalgesia.

As noted above, no significant differences in PPT were observed between the groups, whereas significant between-group differences were observed in pain intensity. Our findings differ from those reported by Tesarz et al. [30], who observed significantly lower PPT values in participants who received an IVR intervention during experimentally induced pain. However, in their study, pain was induced using pressure cuff algometry, which may involve different nociceptive pathways than DN. Inter-individual variability in pain responses to different types of nociceptive stimuli has been previously reported [39], with factors such as age and sex influencing these responses [40,41]. Similar differences have also been found in post-needling soreness following deep dry needling, with women reporting greater pain intensity than men [42]. Therefore, differences in IVR effects may be influenced by both the type of pain and the characteristics of the study population [43].

The strength of the present study lies in the characteristics of the IVR experience, which provided an immersive and enriched environment with an elevated level of presence and interactivity. These user experience factors are considered key contributors to the magnitude of pain relief and its underlying mechanisms [44]. Colloca et al. [38] reported that more immersive and pleasant virtual environments not only reduced pain but also decreased arousal through increased parasympathetic activity. In our study, a nature-based IVR environment was used, which has been associated with enhanced endogenous pain modulation and improvements in mood and anxiety [21]. Similar findings have also been reported in cancer patients, who experienced greater calm and distraction during intravenous access when exposed to nature-based VR content [45]. However, no significant differences in pain during the procedure were found by Scates et al. [45]. According to the novel theoretical framework proposed by Smith et al. [21], nature-based environments within IVR may engage brain regions involved in stress, cognition, mood, and autonomic function, all of which are relevant to pain experience. This effect of IVR might prove to be relevant, as DN has been found to result in immediate activation of the sympathetic nervous system [46]. Thus, nature-based IVR may reduce pain during DN through mechanisms related to attentional distraction and autonomic modulation. However, these mechanisms remain speculative and should be explored in future studies.

From a clinical perspective, preoperative anxiety, which is estimated to affect between 30% and 50% of patients, and fear of needles, with a prevalence rate of 20–30%, are common barriers for people undergoing acute, painful procedures [47,48]. The use of IVR during minimally invasive procedures may help reduce procedural pain, anxiety, and needle-related fear and potentially decrease the need for analgesics or opioids, which can reduce healthcare costs. Thus, patients who would otherwise avoid such procedures may be more likely to undergo them. The benefits of IVR for the healthcare system hold great potential for its implementation in the management of procedural pain. Nevertheless, because this study did not include measures of anxiety, fear, or expectancy, these potential effects remain speculative and should be addressed in future research.

### Study Limitations and Future Studies

While this study makes significant contributions, it is important to acknowledge several limitations that warrant consideration. First, the sample of pain-free young participants may limit the generalizability of the results, particularly in clinical populations such as individuals with chronic pain, myofascial pain syndrome, or needle-related fear. Previous studies have shown that different VR contexts can produce different effects on pain experience [38,49]; thus, transferring our results to other types of VR-based experiences should be done with caution. With respect to the procedures, the number of local twitch responses elicited during the dry needling procedure was not recorded, which limits the interpretation of its potential effects. Previous exposure of participants to dry needling or virtual reality was not assessed. Additionally, no reliability testing of the assessment procedures was performed. Since the control group only received the DN intervention, some components of the IVR group, such as the nature-based IVR environment, music, guided breathing, visual feedback, or novelty, may have significantly influenced the differences between groups. Future studies should incorporate an evaluation of the level of presence within an immersive environment. Additionally, the potential placebo effect of the IVR intervention on pain relief was not explored in this study. Finally, other possible influences, such as anxiety or fear, as well as other potential mediators of pain reduction, such as distraction, relaxation, breathing, expectancy, or novelty effects of IVR during DN, were not examined in our study. Future studies should include comparator groups, such as sham VR, non-immersive nature video, or breathing-only control conditions, for a more rigorous evaluation of placebo-related effects.

Future studies should examine the mechanisms underpinning nature-based IVR during acute procedural pain associated with DN, including neuroimaging, autonomic, psychophysical, and physiological responses to DN. Moreover, further randomized controlled studies with between-group designs, appropriate blinding, and larger sample sizes are needed to establish more robust evidence of the effectiveness of IVR in the management of pain, fear, and anxiety during DN. Finally, future studies should aim to translate these findings into clinical practice and further investigate the potential role of IVR within healthcare systems as an adjunctive, non-pharmacological strategy for procedural pain management.

## 5. Conclusions

This study is the first to examine the immediate and short-term effects of IVR applied during DN. Our findings provide preliminary evidence that IVR may reduce acute procedural pain associated with DN. However, the differences between self-reported pain and PPT call for future studies to understand the mechanisms underlying the IVR-induced hypoalgesia. Furthermore, the effectiveness of nature-based IVR distraction during DN in clinical populations remains to be established.

## Figures and Tables

**Table 1:** Pain intensity values by moment of measurement and group *.

	Immediately Post-DN	1 h Post-DN	6 h Post-DN	24 h Later
DN group	6 (1.6); 5.3–6.7	2.5 (1.4); 1.8–3.1	1.3 (1.3); 0.6–1.9	0.6 (0.6); 0.3–0.9
DN + IVR group	4.6 (1.9); 3.7–5.6	2.4 (2.3); 1.3–3.6	1.4 (1.3); 0.8–2.1	0.3 (0.5); 0.1–0.6

DN: dry needling; DN + VR: dry needling combined with immersive virtual reality. * Values of puncture pain perception are expressed as means (SD) and 95% CI.

**Table 2:** Pressure pain threshold values by moment of measurement and group *.

	Pre-DN	Immediately Post-DN	30 min Post-DN	1 h Post-DN
DN group	4.41 (1.03); 3.67–4.61	3.90 (2.18); 2.90–4.89	3.58 (1.98); 2.68–4.48	3.19 (1.64); 2.45–3.94
DN + IVR group	4.36 (2.17); 3.32–5.41	3.94 (2.24); 2.86–5.02	3.88 (2.48); 2.68–5.07	3.62 (2.38); 2.48–4.77

DN: dry needling; DN + IVR: dry needling combined with immersive virtual reality. * Values of pressure pain threshold (kg/cm^2^) are expressed as means (SD) and 95% CI.

## Data Availability

The datasets generated and analyzed during the current study are not publicly available but are available from the corresponding author upon reasonable request.

## Supplementary Materials

The following supporting information can be downloaded at: https://www.mdpi.com/article/10.3390/jcm15145683/s1, CONSORT 2025 checklist of information to include when reporting a randomised trial.

## Abbreviations

The following abbreviations are used in this manuscript:

IVR	Immersive virtual reality
DN	Dry needling
PPT	Pressure pain threshold
MPS	Myofascial pain syndrome
MTP	Myofascial trigger point
NRS	Numerical rating scale
HMD	Head-mounted display
DN + IVR	Dry needling and immersive virtual reality
MCID	Minimum clinically important difference.
CPM	Conditioned pain modulation
